# How Does Environmental and Occupational Exposure Contribute to Carcinogenesis in Genitourinary and Lung Cancers?

**DOI:** 10.3390/cancers15102836

**Published:** 2023-05-19

**Authors:** Massimiliano Cani, Fabio Turco, Simona Butticè, Ursula Maria Vogl, Consuelo Buttigliero, Silvia Novello, Enrica Capelletto

**Affiliations:** 1 Oncology Unit, Department of Oncology, University of Turin, S. Luigi Gonzaga Hospital, 10043 Orbassano, Italy; massimiliano.cani@unito.it (M.C.); fabio.turco523@edu.unito.it (F.T.); consuelo.buttigliero@unito.it (C.B.); enrica.capelletto@gmail.com (E.C.); 2 Oncology Institute of Southern Switzerland (IOSI), Ente Ospedaliero Cantonale (EOC), 6500 Bellinzona, Switzerland

**Keywords:** lung cancer, genitourinary cancer, environmental exposure, occupational exposure

## Abstract

**Simple Summary:**

Environmental and occupational exposures have been historically related with an increased risk of several diseases and, among these, different cancers. Up to date, neoplasms are among the leading causes of deaths worldwide, and a great knowledge of the related environmental and occupational risk factors is essential to prevent cancer occurrence. Lung and genitourinary cancers account for the highest rates of incidence and prevalence worldwide with a great social and economic impact; thus, we decided to focus on the common environmental and occupational exposures correlated to both lung and genitourinary cancers in order to find any contributions to a decrease in their occurrence or morbidity.

**Abstract:**

Environmental and occupational exposures have been associated with an increased risk of different types of cancers, although the exact mechanisms of higher carcinogenesis risk are not always well understood. Lung cancer is the leading cause of global cancer mortality, and, also, genitourinary neoplasms are among the main causes of cancer-related deaths in Western countries. The purpose of this review is to describe the main environmental and occupational factors that increase the risk of developing lung and genitourinary cancers and to investigate carcinogenesis mechanisms that link these agents to cancer onset. Further objectives are to identify methods for the prevention or the early detection of carcinogenic agents and, therefore, to reduce the risk of developing these cancers or to detect them at earlier stages.

## 1. Introduction

According to the World Health Organization (WHO) estimates in 2019, cancer is one of the leading causes of death in almost 112 out of 183 countries [1]. In most cases, cancer etiology is multifactorial: different elements, as well as genetic predisposition or exposure to environmental agents, can play a crucial role [2]. Risk factors such as age, sex, and family history are actually intrinsic and not modifiable, meanwhile, others, such as exposure to environmental or occupational agents or lifestyle (i.e., tobacco habit, obesity, diet, and alcohol consumption) can be partially or totally adjusted [3,4].

**Figure 1:**
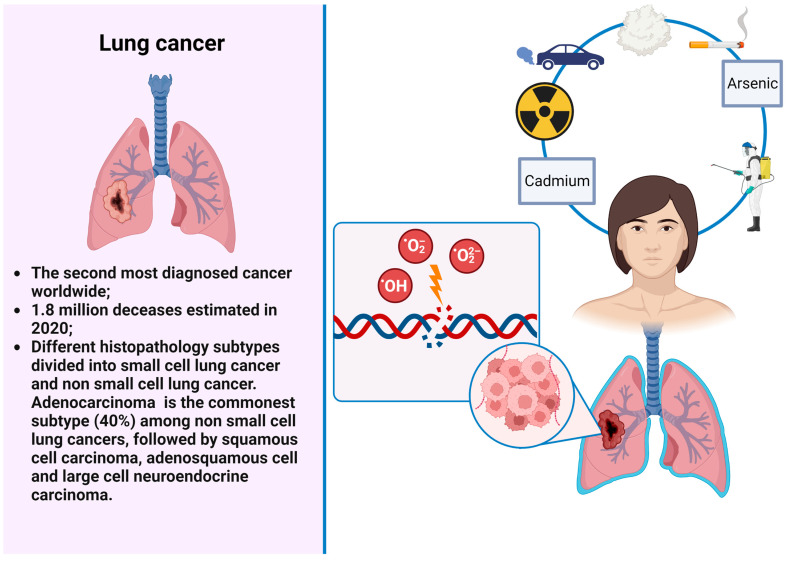
LC and environmental risks factors.

**Figure 2:**
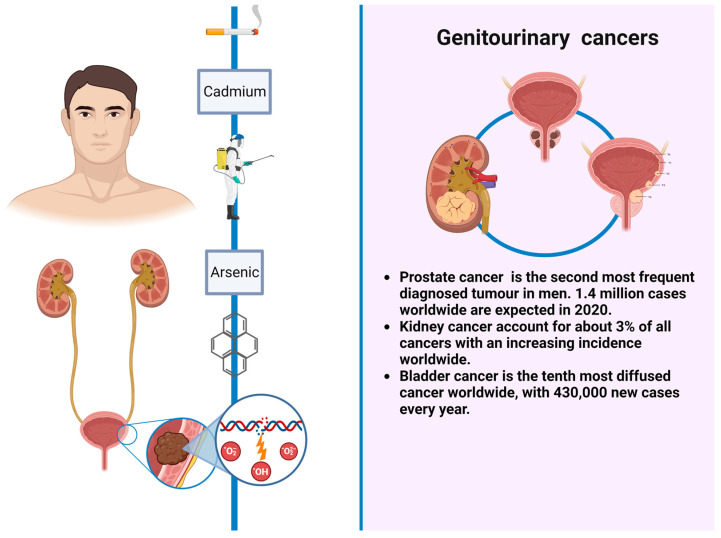
GUCs and environmental risks factors.

Some agents, such as tobacco smoking, polycyclic aromatic hydrocarbons (PAHs), and arsenic, may predispose simultaneously to different neoplasms, in particular, lung (LC) and genitourinary (GUC) cancers (Figure 1 and Figure 2). These are the neoplasms with the highest incidence and mortality worldwide. More particularly, LC is the second most diagnosed cancer and the leading cause of cancer related deaths with an estimated 1.8 million deaths in 2020 [1,5]. LC encompasses different histopathology subtypes, historically classified into small cell lung cancer (SCLC) and non-small-cell lung cancer (NSCLC). Among NSCLCs, adenocarcinoma is the most common subtype (40%), followed by squamous cell or adenosquamous carcinoma and large cell neuroendocrine carcinoma. Tobacco smoking is the most relevant risk factor for LC occurrence, being responsible of two-thirds of all cases of LC [6]. Other risk factors comprise environmental air pollution or exposure to different agents such as asbestos, radon, cadmium, PAHs, diesel exhaust, and household smoke.

In regard to GUCs, prostate cancer is the second most frequent diagnosed tumor in men, with an estimated 1.4 million cases worldwide in 2020 [7]. Different risk factors have been taken into account in prostate cancer occurrence, such as arsenic, cadmium, or pesticides exposure. Renal cell carcinoma represents only 3% of all cancers, with the highest incidence occurring in Western countries. Established risk factors include cigarette smoking, obesity, and hypertension. In the last two decades, renal cell carcinoma incidence has been increasing by approximately 2–4% per year both for a higher rate of incidental tumors and probably for exposure to environmental and occupational cancerogenic factors that nowadays are only partially known [8]. Urothelial neoplasms comprise upper tract urothelial cancers such as renal pelvis and ureter neoplasms and lower tract tumors as bladder cancers; these are the most frequent in this category, being the ninth most diffused, most common cancer worldwide, with 430,000 new cases every year. Tobacco smoking is the most well-established risk factor for bladder cancer, accounting for 50–65% and 20–30% of cases among men and women, respectively [9]. Other risk factors include aromatic amines, PAHs, arsenic, and diesel exhaust exposure [10,11,12,13]. Upper tract urothelial cancers share almost the same risk factors of bladder cancers and, despite the common histological origin, behave differently, being more aggressive and frequently diagnosed at an advanced stage [10,11,12,13,14].

As previously reported, many substances we encounter in our daily life can be related to cancer occurrence. The International Agency for Research on Cancer (IARC) evaluated more than 880 unsafe substances, classifying almost 200 of them as carcinogenic or probably carcinogenic agents, with a large proportion found in occupational settings [15]. IARC described four main groups on the basis of existing scientific data to assess their carcinogenic potential. Details are listed in Table 1.

Once we established the strong influence on tumorigenesis of some environmental and work substances, we decided to review those highly correlated to both LC and GUCs, discussing when available, the most recent epidemiological data focusing on common aspects. We will discuss their cancerogenic mechanisms and how they can influence cancer development. Finally, we will focus on personalized early diagnosis methods (screening programs).

## 2. Tobacco Smoking

Even if tobacco smoking cannot be classified as an environmental or an occupational cancerogenic agent, we decided to include it in our analysis due to its impactful role on both lung and genitourinary cancer occurrences.

Cigarette smoking has a significant impact on public health. In addition to the aforementioned role in several cancers, being otherwise classified in the group one carcinogens by the IARC, it has been linked to an increased disability rate and to several chronic conditions as cardiovascular, pneumological, endocrine, periodontal, or musculoskeletal diseases and, more in general, to 10 years reduced life expectancy [16].

Up to date, almost 60 different tobacco-smoking-related compounds have been taken into account in playing a role in tumorigenesis [17]. Among these, nitrosamine such as 4-(methylnitrosamino)-1-(3-pyridyl)-1-butanone (NNK) and N’-nitrosonornicotine (NNN), polycyclic aromatic hydrocarbons, arylamines, particulate matter, and volatile organics should be mentioned, due to their impactful role in cancer occurrence [18]. In this regard, for example, PAHs interacting with nucleic acids lead to DNA adducts in specific genes such as p53 and KRAS, both correlated to cancer onset. PAHs are also implicated in reactive oxygen species (ROS) synthesis, highly related to cell inflammation and, again, with cancer occurrence. Similar mechanisms are otherwise shared by nitrosamine and volatile organics. Cadmium, also present in cigarette smoking, can act by inhibiting DNA repair mechanisms and by increasing cell-methylation and ROS production, thus facilitating carcinogenesis. All these mechanisms are discussed in more detail in the following parts of our work [19,20,21].

In what regards tobacco diffusion among general population, it was globally estimated that there were 1.14 billion active smokers in 2019 [6]. Although, over the last past decades, tobacco consumption changed: in Western countries, for instance, a consistent reduction in smokers thanks to the adoption of anti-tobacco public health measures was reported, even if an increased incidence has been described among women and the youth with a wide spread of new tobacco products. These latter conditions probably could be related to some observed changes in cancer histologies, for instance, with a lower incidence of squamous cell carcinoma and a higher percentage of adenocarcinoma for LC [22,23]. On the contrary, in low-income countries, tobacco smoking epidemy is still increasing, presenting a consistent public health issue [24].

Over the years, several studies confirmed the strong correlation between LC and the number of cigarettes smoked per day and the smoking years amount, highlighting an increased mortality of two–three times among middle-aged smokers (30 to 69 years old) compared to never smokers [23]. In 2004, Crispo et al., in a multicenter international case–control study, confirmed the role of cigarette smoking on LC onset, finding a cumulative risk of 14–16% for developing LC by age 75 for continuing smokers (at least 1 cigarette per day for at least 1 year) and of 20–25% for heavy smokers (at least 25 cigarettes per day) [25].

Due to the established tobacco-related morbidities, a program of tobacco cessation should be always considered, even after a cancer diagnosis, due to the several benefits of quitting smoking. This has been evaluated by the US Surgeon General’s Report for 2020, which established an improved survival and more in general significant benefits to non-cancer-related health outcomes after tobacco quitting, even for cancer patients. These data were, furthermore, confirmed by a recent meta-analysis of Caimi et al.: by analyzing 21 studies, they found an improved overall survival (OS) for patients who quitted smoking around a LC diagnosis with a summary relative risk (RR) of 0.77 (95% CI: 0.66–0.90) for NSCLC and 0.75 (95% CI: 0.57–0.99) for SCLC [26].

Even considering the strong evidence, in clinical practice, an appropriate smoking cessation program is not uniformly offered to patients at the time of a cancer diagnosis. This is the reason why the International Association for the Study of Lung Cancer (IASLC) has recently released a position statement in this regard. In particular, it recommends the evaluation of patient’s smoking status at the time of diagnosis and, whenever necessary, the integration of a smoking cessation program to all the other cancer cares and treatments [27].

Due to the carcinogens released by tobacco smoking, it is, nowadays, a well-established risk factor also for urothelial cancers (both bladder and upper tract urothelial cancers) and renal cell cancer, being responsible for a 2.5 times higher risk [28,29,30]. This higher risk is also influenced by the number of daily cigarettes and smoking duration without any differences between men and women [31,32]. The estimated latency after smoking initiation and bladder and upper tract urothelial cancers onset is around 20–30 years, and this can explain the latency rates of incidence compared to past smoking prevalence in the general population. Similarly to LC, after smoking cessation, the risk of bladder and upper tract urothelial cancers decreases by around 30%; in this regard, a relative benefit that patients already diagnosed with urothelial cancers may achieve in terms of cancer recurrence and time to progression in case of smoking cessation was also found [33,34]. The role of tobacco smoking in prostate cancer is much more elusive [35], even if recent meta-analyses and systematic reviews have reported a correlation between a worst prognosis for prostate cancer patients with a history of tobacco assumption, in terms of tumor volume, extra-capsular extension, and mortality [36]. Different mechanisms can explain this worse trend, including inflammation and CpG hypermethylation, both highly correlated to tobacco smoking and prostate cancer [37,38].

Second hand smoke, classified as class one carcinogen both in side stream and exhaled mainstream forms, has historically been associated with LC onset when it was observed as a major risk of LC diagnosis in smokers’ wives already in 1981 [39,40]. Similar results were achieved by a comprehensive meta-analysis conducted in 2007 and based on 55 studies; a 27% higher risk of LC was observed for women exposed to second hand smoke [17]. Evidence about the correlation of second hand smoke and urothelial cancers are much weaker, with a great heterogeneity between different studies, probably due to dose exposure differences, and thus more studies are required [41,42].

Finally, considering the unclear data on tobacco-derived products, such as e-cigarettes, heat-not-burn tobacco products, or devices with nicotine alone, it was decided not to include them in this review, as their impact on human health has not been fully elucidated.

## 3. Indoor Air Pollution

Indoor air pollution still represents an important issue, due to the role of many cancerogenic substances released during domestic activities. Sources of pollution can be different such as indoor tobacco active smoking, secondhand smoke, and other gas emissions mostly linked to heating methods, lifestyles, and cooking habits above all in low-income countries, due to the higher amount of time spent in cooking places and the poor residential conditions that prevent proper ventilation.

**Radon**. Radon, an inert gas produced by the natural decay of uranium, due to its wide diffusion as a component of indoor pollution, has been studied and correlated to LC onset and has been considered the main risk factor after cigarette smoke, being, in this sense, classified as a group one carcinogen by the IARC [43,44]. In detail, the alpha particles emitted by Radon decay products such as Polonium-218 and Polonium-241, if inhaled, have been put in correlation with a cancerogenic effect by DNA base mutations and chromosomal strand breaks [39]. In this regard, different studies found a linear correlation between LC risk and Radon exposure, and a recent systemic review has confirmed these facts, even if data heterogeneity and the low variability of Radon exposure did not reach solid conclusions [45,46,47,48]. In order to reinforce the scientific evidence of these data, a multicenter case–control hospital-based study was conducted in Galicia and Asturias, regions of Spain historically considered Radon-prone areas [49]. Including never-smokers exposed to residential Radon proven by specific devices delivered by the investigator team, a higher risk of developing LC in case of Radon indoor levels > 200 Bq·m^−3^ was found, which was, therefore, two times higher than <100 Bq·m^−3^ exposure, with an odds ratio (OR) of 2.33 (95% CI, 1.40–3.89). This trend resulted similar in women and in people who spent more than 20 years in the same residence. Noteworthy, an interaction effect between Radon (>200 Bq·m^−3^) and tobacco smoking exposure was demonstrated, with an OR of 2.75 (95% CI 1.44–5.25) [43]. More recently, different studies correlated Radon exposure to specific mutational patterns. In this regard, the BioRadon France study demonstrated a higher rate of LC among never smokers in French Radon-prone areas, thus assuming a possible role of specific driver mutations [50]. On these bases, a multicenter European trial was designed with the aim of defining the possible correlation between molecular cancer alterations and Radon exposure [51]. Evidence between Radon exposure and cancers onset different than LC are otherwise weak. A Spanish study revealed a higher risk for breast cancer and prostate cancer with a Hazard ratio (HR), in this latter case, of 2.0 (95% CI: 0.9–4.1) in the case of >50 Bq·m^−3^ Radon levels [52]. Similarly, inconclusive results were achieved for kidney cancer or other GUCs [53]. Taking into account the scientific evidence, Radon exposure reduction measures should be promoted through the 2013/59 EURATOM European directive and by increasing the awareness about the possible synergic effect with tobacco smoking.

**Coal and wood indoor combustion**. Several studies investigated the toxic effects of coal-exposed workers (i.e., miners), finding, in this sense, unclear data about the correlation of LC occurrence [54]. In our review, we decided otherwise to focus on coal and wood combustion products in indoor settings, given the importance of this topic and its epidemiological implications. Residential pollutants can vary on people’s habits and lifestyles: as an example, Asian populations tend to use more coal for their residential activities such as heating or cooking than Europeans, who are more used to handling wood for such activities. Both substances and their combustion products (PAHs and PM2.5) have otherwise been related to LC onset, and coal emissions are considered as class one cancerogenic agents by the IARC [55]. In this regard, a recent pooled analysis of seven studies conducted in Asia, Europe, and North America, with a total of 11,689 people enrolled, has confirmed their cancerogenic effects. The risk of LC onset in people exposed to coal combustion products resulted higher, with an OR of 4.93 (95% CI: 3.73–6.52); similarly, in the case of wood combustion, the OR related to LC onset was 1.21 (95% CI: 1.06–1.38) [56]. In the same setting, a recent Chinese study has demonstrated a recurrent molecular pattern of coal-combustion-exposed people, finding out a higher frequency of the ALK+ fusion gene (16.2% versus 8.7% of no-exposed people) and TP53 mutations [57].

**Cooking fumes**. Several studies about cooking oil have demonstrated the release both in food and fumes of several substances that act as carcinogens (i.e., PAHs) [58]. The majority of studies have been conducted on non-smoking Asian women, due to higher LC prevalence in this non-tobacco-addicted population, in order to find the possible correlations among cooking habits [59,60]. In this regard, these data have been recently reinforced by a large study conducted in Taiwan on non-smoking Han Chinese women and was based on 1302 lung cancer cases and 1302 healthy controls. Exposure to cooking fumes was evaluated by a specific index identified as “cooking time years”. A dose-dependent increased risk was demonstrated, with an OR of 3.17 (95% CI: 1.34–7.68), for a cooking time years value > 160. The study also evaluated the cooking type, identifying the pan-frying method as the highest correlated to LC onset, with an OR of 1.53 (95% CI: 1.23–1.89) [61].

The association between indoor pollution and GUCs is less clear, and the heterogeneity of existing data cannot lead to strong conclusions. Ganesan et al., in 2017, tried to correlate repeatedly heated cooking oils with several tumors, including prostate cancer, finding a correlation only for pan-fried foods intake and probably due to glycation end products (AGEs) released by high temperatures. These increasing oxidative stresses play a role on inflammation, and, by interacting with their receptors, can drive prostate cancer onset [62,63].

## 4. Outdoor Air Pollution

Air pollution has been recognized as a certain risk factor for the onset of LC. In this regard, IARC in 2013 classified air pollution and particulate matter (PM) as group one carcinogens [64]. Several studies pointed out this correlation, both in developed countries and in low-income ones [65,66,67]. In 2002, Pope et al., stated that an increase of 10 µg/m^3^ of PM2.5 was associated with an 8% major risk of LC, together with sulfur oxide, considered as another possible cancerogenic agent [68]. Another large prospective Japanese cohort study obtained similar results: it was observed that an increase of 10-units of PM2.5, SO_2_, and NO_2_ were associated with a higher risk of cancer with HR, respectively, of 1.24 (95% CI: 1.12–1.7), 1.26 (1.07–1.48), and 1.17 (1.10–1.26) [69]. Additionally, Gharibvand et al., confirmed this association with an increased incidence of LC every 10 μg/m^3^ of PM2.5 in both never and current smokers [70]. Other interesting and more robust data emerged from a large prospective study, which involved 36 European urban areas and 17 cohort studies from the European Study of Cohorts for Air Pollution Effects (ESCAPE). Air pollution was studied using land-use regression models, considering different types of exposure: PM with diameter less than 10 µm (PM10), 2.5 µm (PM2.5), between 10 and 2.5 (PMcoarse), soot (PM2.5absorbance), NOX, NO_2_, and two different traffic indexes. A total of 312,944 members of different cohorts was included, and, during the follow-up (12.8 years), 2095 were diagnosed with LC. The study revealed an association between PM exposure and the risk of developing LC, showing a risk statistically significant for PM10 concentration with an HR of 1.22 (95% CI 1.03–1.45) per 10 μg/m^3^, and for PM2.5 with an HR of 1.18 (95% CI 0.96–1.46) per 5 μg/m^3^. No association was found for NO_2_, NOX exposure, and traffic intensity at the nearest street, even if a level of 4000 vehicle km/day within 100 m of the residence was linked to an HR of 1.09 (95% CI 0.99–1.21) for LC risk. Among LC histologies, PM10 and PM2.5 exposure resulted in a statistically significant association with adenocarcinoma, not differing considering age, sex, tobacco smoking, fruit intake, or education level. This association resulted also stronger, considering people who lived in the same residence during the follow-up [71]. Similar results were obtained in a previous retrospective study, which, considering China, Mongolia, Japan, and the Democratic People’s Republic of Korea, found a correlation between PM2.5 exposure and LC occurrence [72]. Even if several studies have pointed out the possible correlation between LC onset and air pollution, the molecular mechanisms remained unclear. Recently, at the ESMO 2022 Conference, Swanton and colleagues presented interesting data about the likely correlation between air pollution and the onset of EGFRm lung adenocarcinoma in non-smokers exposed to PM2.5. In particular, the study included 447,932 individuals in South Korea, England, and Taiwan, and authors analyzed normal lung tissue in humans and mice, evaluating, meanwhile, the effects of PM exposure in mice LC models. More specifically, 15% and 53% of normal lung tissue harbored, respectively, EGFR and KRAS mutations in the absence of malignancy, and, when exposed to PM2.5, carcinogenesis appeared more frequently. This could be linked to a macrophage response mechanism and a progenitor-like state in lung epithelium, harboring EGFR mutations, and the possible molecular pathway involved is IL1-b axis [73].

If, nowadays, the evidence between air pollution and LC has become increasingly solid, less is known about the correlation with other solid neoplasms. Inconsistent and heterogeneous data exist about the association with prostate cancer, even if a recent Canadian study based on an historic series found an association between 1420 prostate cancer cases and air pollutants such as PM2.5 and NO_2_. This can be explained by the possible positive correlation between air pollution and levels of AAs or PAHs, which have been already described as cancerogenic substances for prostate cancer onset [74].

## 5. Asbestos

Asbestos fibers belong to natural fibrous silicate minerals and can be found naturally in two different states: serpentine and amphibole forms. Due to their characteristics of resistance to heat and friction, their insulating capacity, and tensile strength, asbestos fibers were widely used in different productive and building processes, leading to a worldwide diffusion. Although, from the 1930s, asbestos tumorigenicity appeared gradually clear, leading later to its classification as a class one carcinogenic agent by the IARC, with an established role for LC, larynx, mesothelium, and ovarian cancer occurrences and to its gradual disuse [75]. Nowadays, we are facing the effects of past exposure, diagnosing cancers directly correlated to asbestos such as malignant mesothelioma or LC [76].

All the commercial and most-used types of asbestos, such as crocidolite, amosite, and chrysotile, are related to LC onset. The histo-pathological mechanism relies on chronic inflammation enhanced by inhaled fibers in the lower airways; this by increasing the levels of ROS and free radicals conducts to DNA damage and thus to an altered cell cycle control and, in the end, to tumorigenesis [77,78,79]. Recently, new molecular mechanisms have been claimed and based on epithelial to mesenchymal transition (EMT), driven by the transforming growth factor (TGF) β [80].

Markowitz and colleagues, in a large report that involved 2377 insulating workers, found a strong correlation between asbestos and LC in never smokers, with a RR of 3.6 (CI 95% 1.7–7.6); this correlation resulted otherwise much stronger for smoker workers, with a RR of 14.4 (95% CI: 10.7–19.4), thus providing an additive effect [81]. Tobacco smoking, in fact, seems to act in a synergistic way to asbestos damage, by facilitating genetic alterations, reducing bronchial clearance, and thus easing fibers accumulation [78].

Different meta-analyses have claimed a possible association between asbestos exposure and prostate cancer [82,83]. Due to the statistical incongruences and methodological doubts, this evidence cannot be considered conclusive enough compared to other tumors, and another meta-analysis has claimed and disconfirmed these results [84]. Similar findings were achieved also for renal cell carcinoma by two different meta-analyses, resulting in one of them a RR of 1.1 (95% CI: 1.0–1.3), thus demonstrating a lack of correlation [85,86].

On what regards non-occupational exposure, an interesting meta-analysis was conducted in 2022 by Kwak and colleagues. Thirteen studies were taken into consideration, and exposure was classified into neighborhood, domestic, and household types. By the evaluation of different studies, a pooled risk of 1.48 (95% CI: 1.18–1.86), in the case of neighborhood exposure, was highlighted in those people living near asbestos mines, mills, or factories in which asbestos was implied. The asbestos peculiarity regarded its fibers, which, due to their low weight, could be windblown from mines and mills, reaching surrounding areas in high concentrations. Surprisingly, the co-habitants of asbestos workers did not appear to have a significantly higher risk, even if asbestos fibers directly reached the residential environment, with a pooled risk of 1.04 (CI 95%: 0.85–1.27), thus representing a difference from previous studies. The great evidence suggested by this work highlighted the importance of less and less use of asbestos, as it could affect not only exposed workers but also the surrounding areas [87].

## 6. Cadmium

Cadmium (Cd) is a widespread transition metal found naturally in the Earth’s crust, representing one of the most extensive occupational and environmental pollutants due to its wide use in industrial and agricultural activities [88,89]. Professional exposure is due to the presence of cadmium in suspended dust or in fumes, and the main absorption path is through the airways or by accidental ingestion from contaminated hands and/or through food [88,89]. Moreover, due to high involvement in many industries, cadmium has been diffused in the environment, and thus also non-occupational exposures should be taken into account, i.e., by food or water intake or by cigarette smoking, where cadmium is also present [21]. Cadmium is associated with a wide range of negative effects on human health such as osteoporosis and bone fracture, type 2 diabetes, and kidney and cardiovascular disease. In addition, cadmium has been recognized as a group one human carcinogen by the IARC for its well-established role in LC onset based on smelting workers studies. The cancerogenic effect of cadmium is related to the inhibition of DNA repair mechanisms together with an increased cell-methylation and ROS production due to its interaction with mitochondrial electron transfer chain. This increases cell oxidative stress and the mutational rate and thus the possibility of cell cancerogenic transformation [21]. The inhibition of apoptosis proto-oncogene activations, tumor suppressor gene inactivations, and cell adhesion disruptions seem to be affected too [89]. Some studies have demonstrated that the prostate is a target organ for cadmium deposits, and numerous in vitro and in vivo experimental studies showed that cadmium could act as a prostate carcinogen in rats [88,89]. Cadmium is an endocrine disruptor that acts by blocking signal transduction inside cells, and, in addition, it can exert estrogenic activities, including the proliferation of prostate cells and the activation of estrogen receptor-a. Although, several epidemiologic studies investigated the association between cadmium exposure and susceptibility to prostate cancer, finding only inconsistent results probably due to different cadmium measurements [13]. In this regard, a meta-analysis published in 2016 showed that only high cadmium exposure can be considered as a risk factor for prostate cancer in occupational settings, with an OR of 1.66 (CI 95%: 1.10–2.50) [90]. Similar results were obtained considering LC occurrence. In 2016, Cheng et al., performed a systematic literature review, and, by analyzing 11 articles, authors did not find strong evidence between cadmium exposure in both general and occupational populations. These results were in contrast with previous evidences, even if authors still suggested a possible positive association, as established by other studies [91,92].

## 7. Arsenic

Arsenic (As) is a chemical element highly associated with other metals such as copper, mercury, and gold [93]. Its exposure is related to skin lesions, nervous system disorders, cardiovascular diseases, and myocardial infarctions, and, from the 60s, it has also been correlated to cancers occurrence and is thus considered as class one carcinogen by the IARC with sufficient evidence in LC, malignant-non melanoma, and bladder cancer occurrences [75,88]. How arsenic acts as a cancerogenic agent remained unclear for many years, and, only recently, it was demonstrated as an activity in genomic alterations by affecting DNA repair mechanisms, miRNA modifications, and epigenetic changes. Once absorbed into the gastrointestinal tract, arsenic undergoes a process of reduction from arsenate to arsenite by the antioxidant glutathione. Arsenite is then involved in oxidative methylation, leading to the production of several subspecies that interact and inhibit the mitochondrial complexes I and III, conducting to ROS, reactive nitrogen species (RNS), and free radical synthesis. In this regard, Martinez et al., reported in never-smoker highly exposed to arsenic lung squamous carcinomas, chromosomal deletion at 1q21 locus, and DNA amplifications at the 19q13.31 and 19q13.33 loci as a consequence to arsenic exposure [94,95]. In vitro and in vivo studies confirmed otherwise the role of arsenic in epigenetic modifications with the hypermethylation of the CDKN2A, p53, Ink4/Arf, p16, and RASSF1A genes [95,96,97]. Moreover, arsenic alters several cell pathways such as the PI3K/AKT, EGFR, and Nrf2-KEAP1 pathways [95] and can also affect miRNAs expression (i.e., miR200b or miR143), involving molecules such as p53 or AKT and their targets [97]. In this regard, arsenic can cause the down-regulation of miR-143, resulting in the transformation of prostate epithelial cells into cancerous cells. This phenomenon determines the increased expression of the genes BCL-2 and BCL-XL and of the metallopeptidases MMP-2 and MMP-9 causing, respectively, an increased resistance to cell death and an increased ability to metastasize [98]. In addition, according to Tallae and Waalks, there was clear in vitro evidence of arsenic in leading androgen independence during prostate cancer cell progression [88]. A Taiwanese study showed in this sense that prostate cancer mortality decreased after the improvement of drinking-water supply system by eliminating arsenic ingestion from the artesian well water, and thus the authors claimed a direct cause and effect relationship with arsenic exposure [98]. In this regard, due to its high presence in drinking water, urothelial cancers (both bladder cancer and upper tract urothelial cancer) were also dose-related to arsenic. Steinmaus et al., showed a markedly increased bladder cancer incidence in adults exposed to arsenic in early life, even up to 40 years after high exposures ceased. As already stated, arsenic has been linked to epigenetic effects such as altered DNA methylation, histone modification, and miRNA expression, and these might also increase long-term cancer risk [12]. The kidney is another target for arsenic carcinogenesis, even if, in animal experiments, the evidence for arsenic-induced kidney cancer is still limited. In this regard, there is evidence that a metabolite of arsenic, called dimethylarsinic acid, increases the risk of developing renal cell carcinoma. In addition, prolonged exposure to arsenic can cause an overexpression of hypoxia-inducible factors 2-alpha (HIF2a), which are often present in kidney cancer [8,99]. In recent years, the major concern about arsenic exposure regards the areas surrounding mine facilities due to arsenic spreading in the soil and groundwater. Such a problem seems to affect above all low-income countries such as Bangladesh, where almost 77 million people were chronically exposed to high As levels in water and soil [97,100]. Furthermore, arsenic pollution is also related to the indiscriminate use of pesticides and herbicides, regarding, again, low-income countries [97]. The US Environmental Protection Agency (EPA) in 2006 established a maximum safe concentration of 10 μg/L as the highest level of arsenic in drinking water, and it has been estimated that 200 million people worldwide have been exposed to higher levels [101].

## 8. Chromium

Chromium (Cr) is one of the most prevalent elements in the Earth’s crust. The hexavalent chromium form (CrVI) is used in manufacturing processes such as chromate production, the plating process, or paint pigment production, and it is classified as a class one cancerogenic agent by the IARC, as it is correlated to LC occurrence with sufficient evidence [75]. Respiratory and dermal exposure are the main forms of absorption in the blood stream, and, after its penetration in cells, chromium VI is reduced by NADPH, GSH, and ascorbate in more reactive species such as chromium IV, chromium V, and free radicals [102]. These interact with DNA, inducing adducts and strand breaks, enhancing cancerogenic onsets. Furthermore, methylation seems to be affected as well, and this should be taken into account due to its effects on genomic stability. In this regard, Sun and colleagues proved the silencing of MLH1, a tumor suppressor gene, in in vitro studies on lung cells [103]. Through in vivo and in-cell cultures, a recurrence of genetic events in workers exposed to chromium VI was also showed, such as the loss of MLH1 expression, p16INK4A methylation, and a low percentage of p53 mutations [104] also on urothelial cell lines, thus reinforcing the possible role of chromium in different cancers, such as LC and bladder cancer [102,105].

## 9. Nickel

Nickel (Ni) is a transition metal highly represented in the environment. It can exist in different oxidative states (from −1 to +4), +2 (Ni^2+^) being the most diffused form. Due to its ductal and resistant proprieties, it is massively implied in several metallurgical processes, as well as in nickel-cadmium batteries’ production or food and chemical industries. Apart from natural nickel sources, due to human industry activities, environmental nickel levels hugely increased in water, soil, and air, thus representing a new relevant health issue [106]. In this regard, inhalation is the most significant source of contamination, followed by skin contact or the ingestion of food or drinking water. Nickel can express several toxic effects, consisting in neuro and cardiotoxicity, teratogenicity, lung fibrosis, skin lesions, allergies, and carcinogenicity. Nickel compounds and nickel alloys have been classified, respectively, as group one by the IARC, due to an established risk for LC and nasal cavity/paranasal sinus cancers onset [107,108,109,110]. How nickel acts as a carcinogenic has not been fully understood. Considering inhalation route, nickel insoluble compounds have been correlated to cancer occurrence due to their longer contact with lung epithelial cells, thus inducing DNA damage, as otherwise proven by in vitro models [111]. Epigenetic events have been claimed, hypothesizing that nickel compounds can cause hyperubiquitination, hypermethylation, and hyperphosphorylation [106]. In this regard, by inducing heterochromatinization, nickel can determine a lower expression of tumor-suppressive genes, thus enhancing cancer occurrence [112]. Among the years, several epidemiology studies have confirmed this correlation [108,109,113]: a recent pooled case—control analysis, including 14 studies from SINERGY project and 16,901 LC cases/20,965 controls, evaluated the risk of LC occurrence in nickel-exposed workers. Among men, exposure prevalence resulted in 24% of cases and 19% of controls, with a median nickel exposure of 22.7 μg/m^3^ years for cases and 21.5 μg/m^3^ years for controls. An OR of 1.29 (95% CI: 1.15–1.45) for the highest quartile of cumulative nickel exposure (>78.1 μg/m^3^ years) was identified. Furthermore, a 30-year exposure was correlated to an OR of 1.23 (95% CI: 1.09–1.38) compared to never exposed. These results were then confirmed also among women as well as in subgroup analyses considering smoking status, finding, in this sense, a relevant additive effect [110]. These data confirm other result, such as the ones already achieved in 2019 by Sciannameo et al., In their analysis of 2991 electroplating workers, a proportion of whom resulted from exposure to chromium and nickel, LC was found to be positively related to a high cumulative exposure to nickel compounds with a HR of 5.48 (95% CI: 2.89–10.38) in the case of high levels of exposure [114]. On what regards GUCs, evidence is still weak: nickel exposure has been correlated to both prostate and kidney cancers [115]. A nested case—control study including 59,778 cases of kidney cancer and 298,890 controls established an higher risk for kidney cancer occurrence among high exposed subjects under the age of 59 years old with an OR of 1.49 (95% CI: 1.03–2.17) [116]. More evidence in this regard is otherwise needed, as recent data seem not to confirm this correlation, for prostate cancer above all, probably due to changes in occupational nickel exposure.

## 10. Polycyclic Aromatic Hydrocarbons (PAHs)

Polycyclic aromatic hydrocarbons (PAHs) are organic compounds characterized by the presence of at least two benzene rings in their molecular structure. They are widely diffused in everyday life and can be found in coal or oil deposits or produced by the combustion of multiple organic materials such as tobacco cigarettes. Among PAHs, dibenzo[a,h]anthracene (DBA), 3-methylcholanthrene (MC), 5-methylchrysene, 7,12-dimethylbenz[a]anthracene, and benzo[a]pyrene (BP) are the most relevant. PAHs have been put in correlation with human cancers, and, nowadays, their role in cancer biology has been well established. In this regard, BP has been classified by the IARC as a class one carcinogen due to its capacity of making DNA adducts, leading tumorigenesis with a well-known role in LC [117]. In this regard, BP it is often used as an exposure indicator, and it is related to cigarette smoking, indoor and outdoor air pollution, or food intake [118,119]. Inhalation and skin contact are the main sources of exposure to PAHs and regard occupational activities such as coal processing, roofing, wood impregnation, aluminum production, lubricating oil use, or exposure to engine exhaust emissions [120]. Once absorbed, thanks to their lipophilic nature, PAHs can directly reach cell plasma, being processed by three different pathways with the production of reactive subspecies. In particular, PB is metabolized to 7, 8-diol-9, 10-epoxide (BPDE) after CYP1A1/1B1 and epoxide-hydrolase reactions. BPDE is the main substance, with the real cancerogenic activity thus making PAHs more precisely pro-cancerogenic elements; particularly, reacting with N2 of guanosine, they form DNA adducts in specific genes such as p53 and KRAS, which are highly correlated to cancer onset [19,121,122,123]. PAHs metabolism also leads to the production of ROS by the aryl hydrocarbon receptor (AHR) via, thus increasing tumorigenesis. PAHs binding AHR seem also to activate cell proliferation and inflammation together with cell-loss adhesion, which are crucial in cancer onset and metastasization [124]. Recent preclinical studies suggested a new hypothesis based on a possible influence of PAHs on gene expression via microRNA regulation. It has been shown that Human Bronchial/Epithelial cells (HBE) exposed to PAHs and their metabolites increased the levels of miRNAs involved in cell cycle control. In detail, miR-22 and miR-494 are responsible of a reduced expression of the tumor suppressor gene PTEN; miR-638 suppresses BRCA1 proteins, and miR-106a plays an inhibitory role on apoptosis and cell cycle arrest [125]. As a consequence, all these events can result in facilitating tumorigenesis by the loss of cell cycle control. Both LC and GUCs have been correlated to PAHs: in a pooled analysis, Olsson et al., in 2022, revealed an association between BP exposure (≥0.24 BP μg/m^3^) and LC, especially for small cell and squamous cell histologies with an OR, respectively, of 2.53 (CI 95% 1.28–4.99) and 1.33 (CI 95% 0.8–2.21). Notably, considering the most recent studies included in the series, the authors did not find any association, and this could be probably due to different levels of work exposure thanks to protective systems [118]. On the same topic, in 2014, Rota et al., elaborated on a meta-analysis, which included workers involved in carbon black production, asphalting activities, and aluminum, iron, and steel manufacturing. For iron and steel workers (i.e., in foundries), an excess risk for respiratory tract cancers was found, with a RR of 1.31 (CI 95% 1.08–1.59); otherwise, the association resulted as inferior to aluminum workers, with a RR of 1.08 (CI 95% 0.95–1.23) [126]. As already stated, there is an increased risk of developing bladder cancer in PAH-exposed workers. The risk of bladder cancer due to occupational exposure is significantly greater at least after around a decade of exposure, with a mean latency of 30 years [10]. On the contrary, the correlation between prostate cancer and PAH exposure is weaker, and the current evidence is heterogeneous. Notably, none of them have evaluated the impact of screening measurers or cancer aggressiveness. Of note, Barul et al., in 2021, in a large population study, showed a weak association between wood-derived PAHs’ occupational exposure and prostate cancer, with an OR of 1.06 (95% CI 0.95 to 1.18) [127]. Even if the results of this latter study, some doubts still remain, considering the metabolisms of PAHs are highly correlated to GST polymorphisms (i.e., GSTP1) that have been linked to early phases of prostate carcinogenesis [127].

## 11. Aromatic Amines

Until the end of the 1970s, several aromatic amines (AAs) (i.e., 4-aminobiphenyl, benzidine, 2-naphthylamin, 4-chloro-o-toluidine) were used in hair dyes and other hair products. On the basis of epidemiological and case–control studies, they were later considered as carcinogenic agents, and restrictive laws were adopted in 1978 by banning some AAs from hair dye ingredients [10]. Nowadays, IARC classifies occupational exposure to hair dyes as a probable carcinogen condition (group 2A), especially for bladder cancer, considering, meanwhile, the still-safe personal use (group 3), as confirmed by a recent prospective cohort study by Zhang et al. [15]. In this regard, the meta-analysis of Harling et al., showed an increased and statistically significant risk for bladder cancer onset among hairdressers who were classically exposed to high concentrations of AAs [9,10]. In the regard of LC, AAs seem to have a role in tumorigenesis, and a recent meta-analysis put forward the correlation of N-Acetyltransferase 2 (NAT2), an enzyme involved in the metabolism of xenobiotics as aromatic amines, to LC onset. More in detail, people exposed to AAs resulted in a reduced risk in the case of NAT2 rapid phenotypes compared to slow phenotypes, with an OR of 1.61 (95% CI: 1.07–2.42), confirming the role of AAs in LC occurrence [20].

## 12. Trichloroethylene

Trichloroethylene (TCE) is used primarily in the vapor degreasing of metal parts, dry-cleaning, textiles, health services, agriculture, and electronic and leather processing. Due to public health concerns, from the 1970s, trichloroethylene use in most industries has been phased out, and it has been replaced by other solvents [128]. IARC currently classifies trichloroethylene as a group one human carcinogen [15]. In humans, epidemiological associations of cancer risk resulted strong above all for kidney cancer, as proved by Karami et al., who supported the correlation between occupational trichloroethylene exposure and kidney cancer risk, with a relative risk of 1.32 (95% CI 1.17–1.57) [128]. This, including pesticides, can be related to trichloroethylene metabolism, which leads to intermediated synthesis such as reactive cysteine S-conjugates that can exert nephrotoxic and nephrocarcinogenic effects [8,128]. The association between LC and trichloroethylene has not been proved, and current literature data in this regard are more than lacking. Notably, the possible role of trichloroethylene for other lung disorders such as pulmonary veno-occlusive disease should be mentioned [129].

## 13. Pesticides

Pesticides are chemical substances widely used in different work activities, agriculture being the most related setting. Some of them have shown cancerogenic activity similar to 4,4’-dichlorodiphenyltrichloroethane (DDT) and have been classified as a group 2A carcinogen, due to a related risk for liver and bile duct cancers. Another one is methyl bromide: it is highly effective as a fumigant and is still widely employed today, despite its activity as an atmospheric ozone depletor and its possible tumorigenicity, representing a substantial environmental and human health risk factor [15,130]. Pesticides, and thus methyl bromide, after their absorption by inhalation or skin contact can have the potential to act at different levels, affecting, e.g., genome integrity or interacting with the human endocrine system, behaving as endocrine disruptors [131]. Pesticide metabolism has been claimed to have a role in renal cell cancer occurrence: halogenated alkanes or alkene metabolites once absorbed are bio-activated in the renal parenchyma by glutathione S-transferases (GSTs) [132]. In this context, renal cell cancer risk may increase, due to the activities and expressions of altered GST activity for GST-mu (GSTM1) and theta (GSTT1) polymorphisms [133]. In this regard, Karami et al., confirmed this hypothesis: they found an increased risk of renal cell carcinoma after pesticide exposure, with a linear correlation to duration and cumulative exposure (OR 1.6, 95% CI 1.00–2.55). Moreover, the risk resulted higher among exposed subjects with both GSTM1 and GSTT1 active genotypes (OR: 6.47; 95% CI: 1.82–23.00; P-interaction: 0.02) compared with unexposed subjects with at least one GSTM1 or T1 inactive genotype [132]. As pesticides act as endocrine disruptors, they have been correlated to prostate cancer occurrence [134]. In this regard, Lewis-Mikhael et al., in their systematic review, observed an association between pesticide exposure and prostate cancer, mostly confined to farmers exposed to high levels of pesticides (OR 1.33, 95% CI 1.02–1.63, *p* = 0.024) [131]. Due to their absorption routes, the correlation between LC and pesticide use has been explored, finding, among the years, controversial data [135,136]. Recent findings from systematic reviews and exposure epidemiology studies confirmed this association: Kim et al., by evaluating 7471 subjects, found an HR of 1.82 (95% CI: 1.11–2.98) between LC and occupational pesticide exposure, with a higher rate among men. These data have been also confirmed by considering different propensity score matching (PSM) methods, thus reinforcing the statistical significance of the analysis [137].

## 14. Diesel

Recently, greater occupational exposure to diesel exhaust has been suggested as a significant risk factor for UC [13]. IARC has already classified diesel engine exhausts as carcinogenic agents, including them in group one, due to their well-defined roles in LC occurrence [15]. Chromosomal damage, altered gene expression patterns, and inflammation can, in the end, explain how diesel exhausts act as tumorigenic agents [9,13]. Data from the SINERGY project confirmed and reinforced the correlation with LC risk, finding an OR of 1.31 (95% CI: 1.19–1.43) for a cumulative diesel exhausts exposure [138]. Recently, greater occupational exposure to diesel exhausts has been suggested as a significant risk factor for urothelial cancer [13]. Boffetta et al,. conducted a meta-analysis of diesel exhausts among high exposure occupations, finding a RR for bladder cancer of 1.23 (95% CI, 1.12–1.36) for any exposure and 1.44 (95% CI, 1.18–1.76) in the case of high exposure [13]. Moreover, the findings from two large case–control studies showed a significant positive association between heavy exposure to diesel exhausts and bladder cancer risks, providing further evidence in this setting [24,28].

## 15. Aristolochic Acid

The Aristolochia family of herbaceous plants has been used worldwide for medicinal purposes for more than 2000 years until its toxicity was understood. Aristolochic acid irreversibly injures renal proximal tubules, resulting in chronic tubulointerstitial disease, while the mutagenic properties of this chemical carcinogen lead predominantly to upper tract urothelial cancers, being considered as a group one carcinogen by the IARC [14]. The toxicities of the Aristolochia species were recognized in the 1990s, when about 100 women with the aim of losing weight took Aristolochia fangchi, developing rapid renal failure and, in some of them, upper tract urothelial cancers [139]. This syndrome was called Chinese herbs nephropathy (CHN), and, when DNA adducts formed with aristolochic acid (AA) were detected in the renal tissues of these patients, the pathogenic role of aristolochic acid in such disease was clarified [140]. In fact, aristolochic acid can react with DNA to form pathogenic adducts that can persist for decades, making them useful biomarkers of exposure [141]. These adducts generate a unique mutational spectrum, characterized by A>T transversions located predominately on the non-transcribed strand of DNA.

Subsequently, researchers noted that CHN was similar to another nephropathy present in the Balkans that occurred only in some villages along the Danube River [139], where people were exposed to Aristolochia clematitis, which grows in the wheat fields [140]. This hypothesis was confirmed when, in the kidney of these patients, high levels of aristolactam-DNA adducts were found, thus representing an exposure biomarker [141].

## 16. Ionizing Radiations

Ionizing radiations (IR) have a well-established and clear role in carcinogenesis, being considered a group one carcinogen for several tumors (i.e., LC, salivary gland, esophagus, stomach), as a consequence of genome damage. What remains unclear is the risk of people, in particular, almost all nuclear workers, exposed to protracted low doses of ionizing radiation. In order to answer to this issue, a French, English, and American observational study among nuclear workers was set up (INWORKS) [142]. Data from 308,297 workers were included and linked to death registers. They were all monitored by personal dosimeters in order to establish extra worker exposure. It was found that a death’s estimated excess relative rate per Gray for solid tumors was 0.47 (90% CI: 0.18–0.79) [143]; on what regards site-specific cancer risk, an excess relative rate per Gray for an LC of 0.56 (90% CI: 0.08–1.02) and 0.25 (90% CI: −0.38–0.87) for prostate cancer was demonstrated. LC compared to other cancer sites, therefore, resulted as being more radiosensitive, thus confirming previous data [142]. Apart from work exposure to ionizing radiation, other possible sources rely on radiotherapy (RT) for medical care [144], but, as this topic varies from the intentions of our work, no more data will be discussed.

## 17. Screening Programs

As already discussed, over the years, several efforts have been made to promote screening programs among high-risk subjects (i.e., heavy smokers for LC) or for those tumors highly represented in the general population as breast or prostate cancers. On what regards LC, recent evidences have highlighted the importance of screening programs, proving their effectiveness in different clinical trials. The National Lung Screening Trial (NLST) in the US demonstrated that low-dose CT scan screening on high-risk subjects, such as heavy smokers, was associated with a reduction in LC mortality by 20% (95% CI: 6.8–26.7, *p* = 0.004) [145]. Similar results were also achieved by the European Nederlands–Leuvens Longkanker Screenings Onderzoek (NELSON) trial, with a potential 24% decrease in LC deaths in heavy smokers (95% CI: 0.61–0.94; *p* = 0.01) [146]. A major concern regarded false positive rates and the possibility of over-diagnoses. However, they resulted as similar to those of mammography screening and, more generally, LC screening programs, resulting from cost-effective measures. Based on NLST results, LC screening programs have been recommended by the U.S. Preventive Services Task Force (USPSTF) in December 2013 and were implemented in current guidelines [147]. Nowadays, in the US, annual low-dose chest CT scans are recommended for people aged 50–80 years with a smoking history of 20 pack/years and active or previous smokers who have stopped within 15 years [148]. During the years, other national health systems launched their own screening programs (i.e., UK) or otherwise promoted supplementary clinical trials, with the aim of reinforcing previous results and introducing low-dose CT-scans in basic levels of assistance, as recently suggested by international scientific societies such as the IASLC [149,150].

The efficacy of LC screening has been demonstrated by recent meta-analyses and real-word data. Potter et al., in this regard, reported an increase in stage I NSCLC diagnoses among US subjects aged 55–80 years as a potential result of LC screening [151,152]. Nevertheless, some issues seem to come up, such as the equal access to screening programs for low-income populations (i.e., Hispanic or Black people in the US). This could be related to limited accessibility to screening centers, low awareness, and cost concerns [153]. The recent report from Potter et al. confirmed this trend, demonstrating the higher percentage of stage IV NSCLC diagnoses in this subgroup of patients compared to high-income subjects, probably due to a reduced involvement in screening campaigns [151].

In prostate cancer, the use of PSA as a screening test remains one of the most controversial topics in urological literature. In fact, routine PSA screening, even if it leads to an increase in prostate cancer diagnoses, is associated with a significant risk of over treatment, and, to date, it is not routinely recommended by the guidelines [7]. Additionally, in urothelial carcinoma and renal cell carcinoma, there are no validated screening programs since the studies performed have not demonstrated adequate cost-effective rates.

## 18. Discussion and Future Directions

Approximately 10% of cancers worldwide are strongly related to environmental and occupational factors (as summarized in Table 2) [1]. There is robust evidence that a proportion of LC and urothelial cancers is related to environmental and/or occupational exposures, while, in prostate and renal cell carcinoma instances, the evidence is weaker [4]. These cancers are, therefore, preventable through primary prevention measures such as environmental decontamination, the ban of cancerogenic substances, or the promotion of awareness campaigns about healthy lifestyles or smoking cessation [154,155].

On what regards tobacco smoking in terms of global tobacco control policies, the WHO promoted a group of measures from 2008 that were addressed to contrast cigarette smoking, the so-called MPOWER package. It consisted of six effective public health strategies to monitor and prevent tobacco diffusion, protect people from tobacco smoking, offer proper programs to encourage tobacco quitting, warn about the negative effects of tobacco smoking, enforce all strategies to ban tobacco product advertising, and, finally, raise tobacco taxes (MPOWER). Over the years, substantial results have been achieved thanks to this initiative: 65% of the global population was covered in 2018 by one or more MPOWER package measure, and the prevalence of tobacco smoking decreased in almost 116 countries, particularly in those where MPOWER’s policies were implemented [156].

Unfortunately, similar results were not always achievable, especially when public prevention strategies had to face with agents widely present in the environment and were difficult to remove or replace with less dangerous ones. This is the reason why secondary prevention measures are essential. They include strategies for early diagnoses by the use of screening tests in order to improve survival rates in cases of cancer diagnosis.

Screening programs as teachable moments should also offer specific measures to contrast modifiable risk factors correlated to cancer onset. This is true, above all, for tobacco smoking, and, in this regard, all the measures aimed to address encouraging tobacco cessation should be concomitantly promoted to LC screening. Several reports have already demonstrated an increase in tobacco quitting rates as a result of screening enrolment: in fact, 22% of subjects involved in LC screening programs quitted smoking 2 months later the first CT scan [157]. Many other possible strategies can be adopted, such as brief counselling sessions or pre-printed informative material deliveries at the time of screening enrolment. However, higher success rates were observed in case of personal or group consultations in a well-structured cessation program with highly trained personnel [158]. In addition, the administration of drugs to counteract nicotine addiction increased successful rates [159]. Studies available in the current literature on this topic are, however, very heterogeneous in terms of modalities, drugs, or devices employed, and, therefore, solid conclusions cannot be established. Thus, further evidence should be achieved in this setting [149,160,161].

Therefore, it is important to identify patients at a higher risk of developing cancer, such as those exposed to environmental and/or occupational carcinogens, and future studies investigating the use of screening tests in this specific population are needed since these patients may be the ones to benefit most from screening programs. However, the planning of these studies remains a challenge since there are considerable difficulties in assessing and quantifying individual exposure to a specific carcinogen and, therefore, in identifying patients most at risk. Furthermore, the development of cancer after exposure to an environmental or occupational agent can occur even after several years, and this, evidently, makes the execution of these studies more complex.

In the end, although considerable progress has been made in understanding the carcinogenic mechanisms of some agents, they are not always well fully understood, thus further studies are needed to investigate these crucial aspects.

**Table 2:** Environmental and occupational risk factors associated with site-specific tumorigenesis with sufficient or limited evidence, according the IARC classification *.

Risk Factor	Pathogenic Mechanisms	Sufficient Evidence	Limited Evidence	
**Tobacco smoking**	DNA adducts among tumor suppressor genes.	Oral cavity, pharynx, esophagus, stomach, colon, rectum, liver, bile duct, pancreas, nasal cavity/paranasal sinus, larynx, lung, uterine cervix, ovary, kidney, renal pelvis and ureter, urinary bladder	Breast, childhood leukemias (parental smoking)	
**Radon**	DNA base mutations and chromosomal strand breaks	Lung	Leukemias	
**Indoor air pollution (coal combustion)**	DNA adducts	Lung	-	
**Outdoor air** **pollution**	Induced oncogene mutations by still unclear molecular mechanisms	Lung	Urinary bladder	
**Asbestos**	Increased ROS synthesis implicated in DNA damage across tumor suppressor genes	Larynx, lung, mesothelium, ovary	Pharynx, stomach, colon, rectum	
**Cadmium**	Induction of oxidative stress and suppression of DNA repair genes. Alterations of DNA methylation	Lung	Prostate, kidney	
**Arsenic**	DNA strand breaks, chromosomal aberrations. Epigenetic alterations	Lung, skin, urinary bladder	Liver, bile duct, prostate, kidney	
**Chromium**	Damage to cellular components, generation of free radicals resulting in DNA damage.	Lung	Nasal cavity and paranasal sinus	
**Nickel**	DNA damages and epigenetic changes	Lung, nasal cavity and paranasal sinus	-	
**PAHs** **(benzopyrene)**	DNA adducts	Lung, bladder, esophagus, liver, lymphoid and hematopoietic tissues	-	
**AAs (benzidine as hair dyes)**	DNA adducts	Urinary bladder	-	
**Trichloroethylene**	DNA adducts synthesis by reactive intermediates	Kidney	Liver, bile duct	
**Pesticides** **(DDT)**	ROS induced synthesis and subsequent DNA and protein damages	-	Liver and bile duct	
**Diesel**	Chromosomal damage, altered gene expression patters, inflammation onset	Lung	Urinary bladder	
**Ionizing** **radiation**	DNA double-strand breaks	Salivary gland, esophagus, stomach, colon, lung, bone, skin, breast, kidney, urinary bladder, brain/central nervous system, thyroid, chronic myeloid and acute lymphocytic leukemia	Rectum, liver, bile duct, pancreas, ovary, prostate, childhood leukemia	
**Aristolochic acid**	Aristolactam—deoxyadenosine adducts synthesis and subsequent tumorigenesis.	Renal pelvis and ureter	-	

PAHs: polycyclic aromatic hydrocarbons; AA: aromatic amines; DDT: dichlorodiphenyltrichloroethane. * List of classifications by cancer sites with sufficient or limited evidence in humans, IARC Monographs Volumes 1–133—Last update: 24 March 2023.

## 19. Conclusions

Lung and genitourinary cancers are among the most frequent cancers worldwide, and a proportion of these may be associated with environmental and occupational factors. Despite greater awareness about the importance of reducing exposure to certain carcinogens, some of them continue to be widespread in the environment and the workplace. The adoption of effective preventive measures against these carcinogens is essential. Furthermore, future studies are needed to better understand the carcinogenic mechanisms of some agents, finding specific biomarker exposures that can help us to identify patients who have been exposed to these factors and, finally, to evaluate whether the use of specific screening studies may be useful in these patients.

## Figures and Tables

**Table 1:** IARC classification system.

Group	Meaning	Examples
**GROUP 1** **Carcinogenic to humans**	Enough evidence for a proven association with human cancer.	Tobacco smoking, outdoor air pollution, alcoholic beverages, asbestos, arsenic, benzene, formaldehyde, engine exhaust and diesel, ionizing radiation, coal as indoor emissions from household, nickel compounds, welding fumes, chromium-VI compounds, aristolochic acid, cadmium, trichloroethylene, radon, aluminum, iron and steel founding, mineral oils, soot, wood dust
**GROUP 2A** **Probably** **carcinogenic to humans**	Limited evidence for an association with human cancer. Sufficient data of cancer in experimental animals.	High temperature frying, red meat, petroleum refining (only occupational exposure), hairdresser or barber (aromatic amines) as occupational exposure, glyphosate, N-nitrosodiethylamine (NDMA), 4,4’-dichlorodiphenyltrichloroethane (DDT)
**GROUP 2B** **Possibly carcinogenic to humans**	Limited data for an association with human cancer, but insufficient evidence of cancer in experimental animals.	Dry cleaning (occupational exposure), magnetic fields (extremely low frequency), styrene, coffee and pickled vegetables
**GROUP 3** **Not classifiable as human** **carcinogens**	Evidence is inadequate in humans and inadequate or limited in animals.	Acrylic acid, chlorinated drinking water, electric field, fluorescent lighting, hair coloring products (personal use)
